# Predictors of compliance in adolescents with type 1 diabetes mellitus

**DOI:** 10.1192/j.eurpsy.2021.673

**Published:** 2021-08-13

**Authors:** L. Pechnikova, Y. Manuylova, A. Ryzhov, E. Zhuykova, E. Sokolova, A. Tkhostov

**Affiliations:** 1 Faculty Of Psychology, Lomonosov MSU, Moscow, Russian Federation; 2 L.s. Vygotsky Institute Of Psychology, Russian State University for the Humanities, Moscow, Russian Federation

**Keywords:** compliance, diabetes, adolescents

## Abstract

**Introduction:**

Non-compliance is a common problem in diabetes despite of the potentially drastic consequences. The study of the factors of compliance in adolescents with diabetes is not only important due to the possible practical implementations in health care, but also may be threated as a model for understanding the age-specific aspects of compliance behaviours.

**Objectives:**

The study was aimed to evaluate various, primary family-related, factors contributing to compliance behaviour.

**Methods:**

Participants: 71 adolescents (f=44, m=27, age: 13-17) with diabetes mellitus type 1, without insulin pump usage, and their mothers. Instruments: compliance was accessed with MMAS and “Degree of compliance” (for 15-17-olders only) scales. Paternal attitudes were assessed by (1) ADOR questionnaire, yielding scores for: Positive interest, directiveness, hostility, autonomy, inconsistency; (2) Family anxiety analysis questionnaire, with scales: guilt, anxiety, tension. Illness attitudes were assessed with the Concerns of the illness progression model questionnaire. Interview data were used to assess such variables as duration of illness, frequency of therapist consultations y etc.

**Results:**

Stepwise regression analysis suggested the best model for compliance being predicted (R2=.203) by family anxiety (beta=-.406, p<.001), duration of illness (beta=-.218, p<.05) and frequency of consultations (beta=.0212, p<.05). For 15-17-olders only compliance was better predicted (R2=.499) by concerns about illness (beta=.876, p<.001), distraction copings (beta=.501, p=0.001), negative thinking (beta=-.421, p<0.02) and frequency of consultations (beta=.274, p<.05).

**Conclusions:**

Low family anxiety, shorter duration, and more frequent contacts with therapist, as well as productive copings, absence of frequent negative thoughts and fantasies about illness contribute to compliance. Negative emotions hamper compliance instead of fostering it.

